# Hand and Oral Hygiene Practices of South Korean Adolescents Before and During the COVID-19 Pandemic

**DOI:** 10.1001/jamanetworkopen.2023.49249

**Published:** 2023-12-26

**Authors:** Jiyeon Oh, Myeongcheol Lee, Hojae Lee, Hwi Yang, Jaeyu Park, Masoud Rahmati, Ai Koyanagi, Lee Smith, Guillaume Fond, Laurent Boyer, Min Seo Kim, Seung Won Lee, Guillermo F. López Sánchez, Elena Dragioti, Ho Geol Woo, Dong Keon Yon

**Affiliations:** 1 Department of Medicine, Kyung Hee University College of Medicine, Seoul, South Korea; 2 Center for Digital Health, Medical Science Research Institute, Kyung Hee University Medical Center, Kyung Hee University College of Medicine, Seoul, South Korea; 3 Department of Regulatory Science, Kyung Hee University, Seoul, South Korea; 4 Department of Physical Education and Sport Sciences, Faculty of Literature and Human Sciences, Lorestan University, Khoramabad, Iran; 5 Department of Physical Education and Sport Sciences, Faculty of Literature and Humanities, Vali-E-Asr University of Rafsanjan, Rafsanjan, Iran; 6 Research and Development Unit, Parc Sanitari Sant Joan de Deu, Barcelona, Spain; 7 Centre for Health, Performance and Wellbeing, Anglia Ruskin University, Cambridge, United Kingdom; 8 CEReSS-Health Service Research and Quality of Life Center, Aix-Marseille University, Marseille, France; 9 Cardiovascular Disease Initiative, Broad Institute of MIT and Harvard, Cambridge, Massachusetts; 10 Department of Precision Medicine, Sungkyunkwan University School of Medicine, Suwon, South Korea; 11 Division of Preventive Medicine and Public Health, Department of Public Health Sciences, School of Medicine, University of Murcia, Murcia, Spain; 12 Pain and Rehabilitation Centre, and Department of Medical and Health Sciences, Linköping University, Linköping, Sweden; 13 Research Laboratory Psychology of Patients, Families, and Health Professionals, Department of Nursing, School of Health Sciences, University of Ioannina, Ioannina, Greece; 14 Department of Neurology, Kyung Hee University Medical Center, Kyung Hee University College of Medicine, Seoul, South Korea; 15 Department of Pediatrics, Kyung Hee University Medical Center, Kyung Hee University College of Medicine, Seoul, South Korea

## Abstract

**Importance:**

Only a few studies have examined the long-term trends of hand and oral hygiene, especially among adolescents.

**Objective:**

To investigate the 15-year trends in frequency of handwashing and toothbrushing and examine the factors associated with hand and oral hygiene, particularly during the COVID-19 pandemic.

**Design, Setting, and Participants:**

This cross-sectional study, performed from January 1, 2008, to December 31, 2022, used general population–based data from 963 644 individuals in a national representative survey (Korea Youth Risk Behavior Web-based Survey [KYRBS]).

**Exposure:**

COVID-19 pandemic.

**Main Outcomes and Measures:**

Trends in hand and oral hygiene practices were measured by how frequently adolescents washed their hands and whether they fulfilled the recommended guidelines for toothbrushing. An interrupted time series analysis using linear and logistic regression models was performed to assess any associations with the COVID-19 pandemic. Hand and oral hygiene behaviors before and during the pandemic in each sociodemographic subgroup were also compared.

**Results:**

In the 963 644 adolescents (495 697 [51.4%] male; mean [range] age, 15.01 [12-18] years) who participated in the KYRBS from 2008 to 2022, a 73.3% (95% CI, 59.4%-97.4%; *P* < .001) immediate increase was seen in overall hand hygiene behavior at the onset of the COVID-19 pandemic compared with the prepandemic period, with a sustained decrease thereafter (β = −0.018; 95% CI, −0.022 to −0.015; *P* < .001). Meanwhile, no immediate increase was observed in terms of overall oral hygiene behavior (0.1%; 95% CI, −0.9% to 1.1%; *P* = .82); however, there was a sustained decrease during the pandemic (β = −0.018; 95% CI, −0.020 to −0.016; *P* < .001). Older age, female sex, nonsmoking status, alcohol use, low household economic level, and poor school performance were significantly associated with poor hand hygiene during the pandemic.

**Conclusions and Relevance:**

In this cross-sectional study of South Korean adolescents, an increase in the prevalence of hand hygiene was observed during the early pandemic; however, this prevalence decreased over time. Meanwhile, the decrease in the prevalence of oral hygiene was more pronounced during the pandemic. This study recommends stronger guidelines for adolescent health coaches, such as teachers or public health advisers, regarding hygiene behaviors, even after the COVID-19 pandemic ends.

## Key Points

**Question:**

How have the long-term trends of hand and oral hygiene among Korean adolescents changed since the COVID-19 outbreak?

**Findings:**

This cross-sectional study of 963 644 Korean adolescents from 2008 to 2022 found an immediate improvement in hand hygiene behavior but a sustained decrease in both hand and oral hygiene practices during the pandemic. Female sex, older age, nonsmoking status, alcohol use, low household economic level, and poor school performance were considered risk factors for poor hand hygiene.

**Meaning:**

This study encourages more effective promotion programs for hand and oral hygiene.

## Teaser

This cross-sectional study examines whether the COVID-19 pandemic was associated with changes in handwashing and toothbrushing frequency among South Korean adolescents.

## Introduction

Since the declaration of the COVID-19 pandemic by the World Health Organization (WHO),^1,2^ further consideration has been given to hygiene practices to prevent exposure to pathogens via hands and saliva. Specifically, frequent handwashing was strongly recommended by health experts during the COVID-19 pandemic.^3^ The WHO provided a guideline that people should wash their hands with soap and running water before and after meals and after using the toilet.^4^ Because of the efforts made by government and health organizations to increase public interest in hand hygiene, people became aware of handwashing during the pandemic.

The American Dental Association (ADA) and WHO recommend brushing of teeth at least twice a day.^5,6^ A previous study^7^ reported that the oral cavity may be a potential site for SARS-CoV-2 viral replication. Therefore, a few studies have explored the association of the COVID-19 pandemic with hand and oral hygiene (handwashing: Denmark [n = 72]^8^ and France [modeling study]^9^; toothbrushing: Nigeria [n = 996]^10^ and multiple countries [n = 14 970]).^11^ These studies showed conflicting results and weak levels of evidence due to the limited small sample sizes, broad age ranges, restricted socioeconomic status, and short observation periods (mostly up to 2020).

There is a need to analyze long-term trends in handwashing and toothbrushing with representative samples and compare tendencies before and during the COVID-19 pandemic. Hence, this study aimed to investigate the 15-year trend in frequency of handwashing and toothbrushing using nationwide, large-scale data collected between 2008 and 2022. We examined whether the COVID-19 pandemic was associated with the frequency of handwashing and toothbrushing practices among South Korean adolescents.

## Methods

### Study Design

A total of 992 702 individuals participated in the survey during a period of 15 years. After excluding participants with missing data, the final sample size for this cross-sectional study was 963 644. We used general population–based data from these 963 644 respondents of the Korea Youth Risk Behavior Web-based Survey (KYRBS). This survey was conducted annually by the Korean Disease Control and Prevention Agency from January 1, 2008, to December 31, 2022, to assess health behaviors among Korean middle and high school students. This survey covered 98.2% of all Korean adolescents aged 12 to 18 years. It has also been established and reviewed by experts from the Ministry of Education and the Ministry of Health and Welfare. The target population of the survey consisted of both public and private schools in all South Korean provinces. Students were asked to complete a questionnaire in a computer laboratory in each school.^12^ Youth aged 12 to 18 years were recruited, and they voluntarily participated in the survey (mean response rate, 95%).^12,13^ The sampling strategy followed 3 steps. First, the population was stratified by 39 regions and level of school education. Second, proportional allocation methods were used for sampling distribution. Third, school and class units were used for stratified cluster sampling, which minimized sampling errors. Weighting was also considered, allowing the sample to represent the entire population of Korean adolescents. The study protocol was approved by Kyung Hee University and the Korean Disease Control and Prevention Agency. All participants provided written informed consent. Our study adhered to the tenets of the Declaration of Helsinki.^14^ This study followed the Strengthening the Reporting of Observational Studies in Epidemiology (STROBE) reporting guideline.

### End Points

We investigated whether the COVID-19 pandemic changed hand and oral hygiene trends. Given that the first confirmed case of COVID-19 in South Korea was reported in January 2020, the years 2020, 2021, and 2022 were regarded as the COVID-19 pandemic era.^15^ The participants were asked to indicate whether they washed their hands with soap in the following circumstances: before meals at school, after using the toilet at school, before meals at home, after using the toilet at home, and after coming in from outside. The response options for each circumstance were always, often, sometimes, and never. We recategorized the participants based on frequency of handwashing into 2 groups as follows: high (those whose response was always in every circumstance) and low. The question used to assess oral hygiene was, “How many times do you brush your teeth a day?” We followed the international guidelines set forth by the ADA and WHO for brushing teeth at least twice per day.^5,6,16^ We divided the respondents into 2 groups: those who brushed their teeth twice or more per day and those who did not. In addition, we compared the trends in hand and oral hygiene behaviors before and during the pandemic in each subgroup (by grade, sex, smoking status, current alcohol use, economic level, highest educational level of parents, and school performance).

### Definitions of Covariates

The study variables included age, sex, grade (7-9 [middle school] and 10-12 [high school]), body mass index (BMI; calculated as weight in kilograms divided by height in meters squared using height, weight, sex, and age in reference to the 2017 Korean National Growth Charts for children and adolescents^17^), smoking status (yes or no), current alcohol use (yes or no), parents’ highest educational level (high school or lower, college or higher, or unknown), school performance (high, middle-high, middle, middle-low, or low), and household economic level (high, middle-high, middle, middle-low, or low).^18^ All variables are based on self-reported data.^12^ Individuals who smoked at least once within the past 30 days were considered current smokers, whereas those who had not smoked for the past 30 days were considered nonsmokers. We defined alcohol consumers as those who consumed alcohol at least 1 day within the past 30 days.^19^

### Statistical Analysis

We analyzed the KYRBS data to investigate the national trends in hand and oral hygiene behaviors during the past 15 years in the context of before and after the pandemic. Interrupted time series analysis was performed using an ordinary least-squares model (linear regression), logistic regression, and estimate of adjusted odds ratio (OR) to investigate immediate and sustained changes in hand and oral hygiene behaviors before and during the pandemic.^20^ We conducted sex and age standardization and adjustment for sex, grade, BMI, current alcohol use, current smoking status, highest educational level of parents, household economic level, and school performance. Furthermore, to compare the latest prepandemic period (2017-2019) with the pandemic period (2020-2022) more thoroughly, adjusted and weighted ORs were derived using binary logistic regression models.^21,22^ In addition, we examined the individual factors associated with hand and oral hygiene practices using binary logistic regression. Weighted ORs with 95% CIs were obtained after adjusting for the covariates. To evaluate the magnitude of risk change before and after the pandemic, the ratio of the ORs (95% CIs) were used.

All analyses were performed using SAS software, version 9.4 (SAS Institute Inc). A 2-sided *P* < .05 was considered statistically significant.

## Results

Among the 963 644 adolescents who participated in the KYRBS from 2008 to 2022, a total of 495 697 (51.4%) were male and 467 947 (48.6%) female. The mean (SD) age of the study population was 15.01 (1.75) years. A total of 51.2% of the participants were in grades 7 to 9 (middle school) and 48.8% were in grades 10 to 12. The baseline characteristics of the adolescents are presented in the Table.

**Table.** Crude Baseline Characteristics of the KYRBS Study Sample, 2008-2022^a^

Characteristic	Prepandemic (2008-2019) (n = 806 212)	Intrapandemic (2020-2022) (n = 157 432)	Total (2008-2022) (N = 963 644)
Age, mean (SD), y	14.99 (1.75)	15.09 (1.74)	15.01 (1.75)
Grade			
7-9 (Middle school)	408 858 (50.7)	84 921 (53.9)	493 779 (51.2)
10-12 (High school)	397 354 (49.3)	72 511 (46.1)	469 865 (48.8)
Sex			
Male	414 488 (51.4)	81 209 (51.6)	495 697 (51.4)
Female	391 724 (48.6)	76 223 (48.4)	467 947 (48.6)
BMI, mean (SD)			
Underweight	64 640 (8.0)	12 699 (8.1)	77 339 (8.0)
Normal	616 778 (76.5)	108 746 (69.1)	725 524 (75.3)
Overweight	65 155 (8.1)	15 648 (9.9)	80 803 (8.4)
Obesity	59 639 (7.4)	20 339 (12.9)	79 978 (8.3)
Smoking			
Yes	62 120 (7.7)	7021 (4.5)	69 141 (7.2)
No	744 092 (92.3)	150 411 (95.5)	894 503 (92.8)
Current alcohol use			
Yes	145 194 (18.0)	17 618 (11.2)	162 812 (16.9)
No	661 018 (82.0)	139 814 (88.8)	800 832 (83.1)
Highest educational level of parents			
Middle school or lower	11 425 (1.4)	335 (0.2)	11 760 (1.2)
High school	206 840 (25.7)	16 809 (10.7)	223 649 (23.2)
College or higher	374 431 (46.4)	71 364 (45.3)	445 795 (46.3)
Unknown	213 516 (26.5)	68 924 (43.8)	282 440 (29.3)
Household economic level			
High	62 746 (7.8)	17 352 (11.0)	80 098 (8.3)
Middle-high	203 064 (25.2)	45 983 (29.2)	249 047 (25.8)
Middle	383 331 (47.6)	75 751 (48.1)	459 082 (47.6)
Middle-low	123 246 (15.3)	15 241 (9.7)	138 487 (14.4)
Low	33 825 (4.2)	3105 (2.0)	36 930 (3.8)
School performance			
High	96 309 (12.0)	20 194 (12.8)	116 503 (12.1)
Middle-high	198 391 (24.6)	39 309 (25.0)	237 700 (24.7)
Middle	225 788 (28.0)	47 931 (30.4)	273 719 (28.4)
Middle-low	195 328 (24.2)	34 964 (22.2)	230 292 (23.9)
Low	90 396 (11.2)	15 034 (9.6)	105 430 (10.9)

Abbreviations: BMI, body mass index (calculated as weight in kilograms divided by height in meters squared); KYRBS, Korea Youth Risk Behavior Web-based Survey.

^a^ Data are presented as number (percentage) of participants unless otherwise indicated.

**Figure 1.**
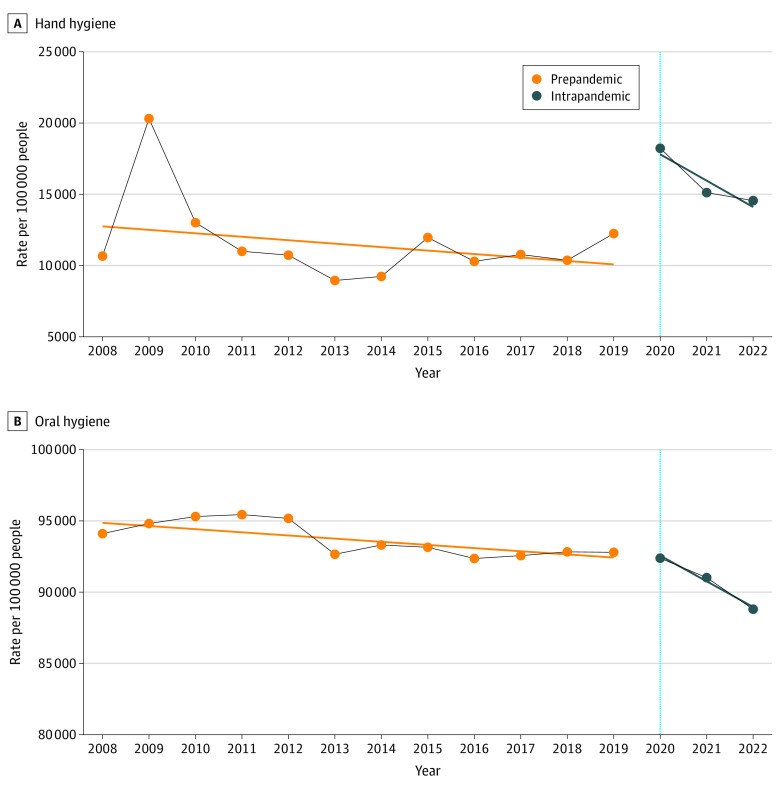
Age- and Sex-Standardized Interrupted Time Series Analysis of Hand and Oral Hygiene Practices and the COVID-19 Pandemic Intervention Among Korean Adolescents, 2008-2022 Data points indicate hygiene prevalence; the straight orange and blue lines are the regression lines of the interruped time series analysis and the vertical line at 2020 indicates the onset of the COVID-19 pandemic.

eTable 1 in Supplement 1 and Figure 1 provide the age- and sex-standardized interrupted time series analyses of hand and oral hygiene practices among Korean adolescents from 2008 to 2022. An immediate increase of 73.3% (95% CI, 59.4% to 97.4%; *P* < .001) was observed in the overall hand hygiene practice at the outbreak of the COVID-19 pandemic; however, it gradually decreased over time (β = −0.018; 95% CI, −0.022 to −0.015%; *P* < .001). Meanwhile, no immediate increase in oral hygiene practice was observed at the onset of the pandemic, with a rate of 0.1% (95% CI, −0.9% to 1.1%; *P* = .82). However, oral hygiene practice showed a sustained decrease (β = −0.018; 95% CI, −0.020 to −0.016; *P* < .001) during the pandemic. We observed similar tendencies for immediate and sustained changes in the subgroups according to grade, sex, smoking, current alcohol use, highest educational level of parents, economic level, and school performance (eTable 1 in Supplement 1).

**Figure 2.**
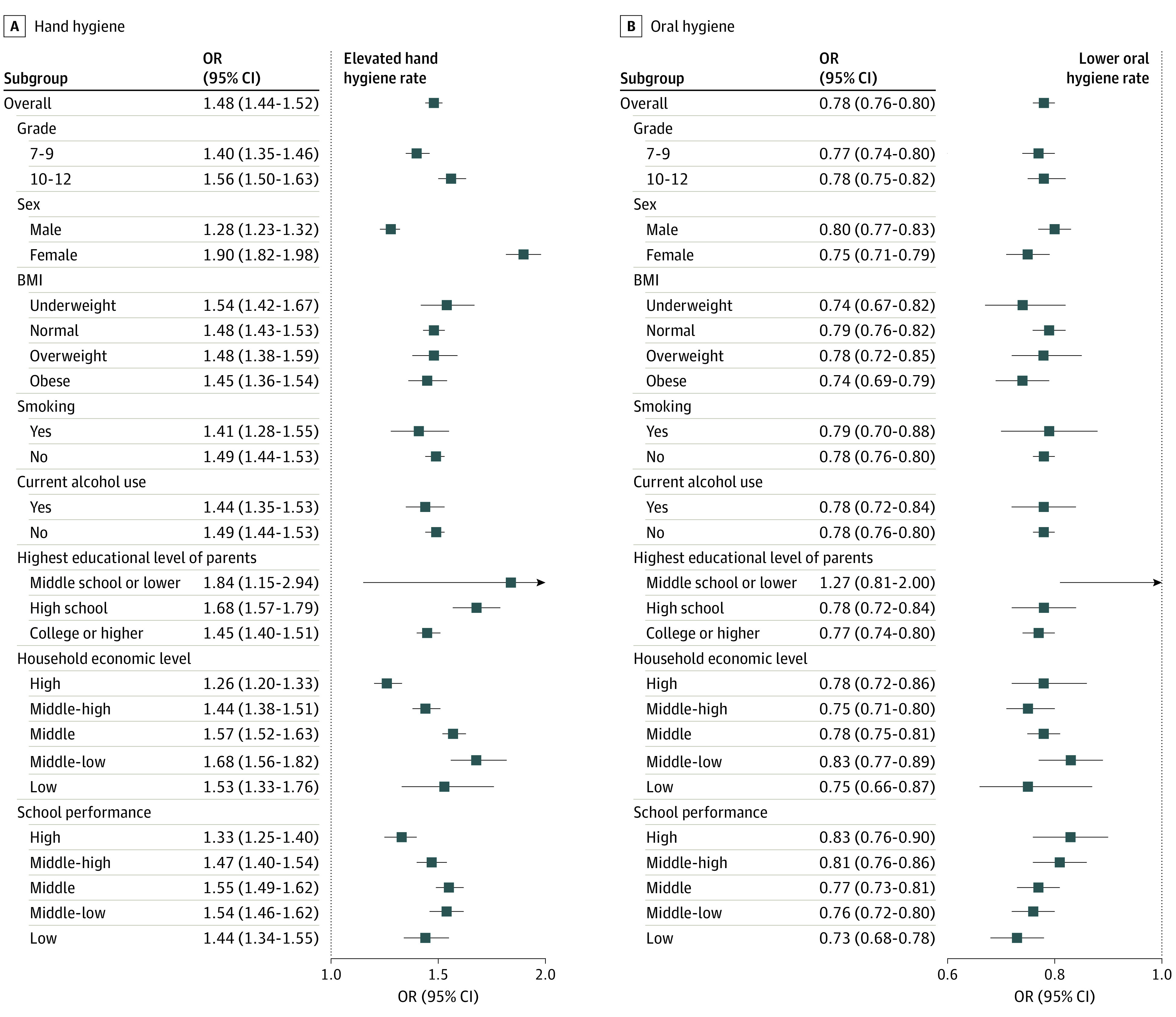
Adjusted and Weighted Odds Ratios (ORs) of Hand and Oral Hygiene Practices, 2020-2022 vs 2017-2019, by Sociodemographic Subgroups BMI indicates body mass index (calculated as weight in kilograms divided by height in meters squared).

Figure 2 shows the adjusted and weighted ORs for the 2020-2022 vs 2017-2019 periods. Elevated hand hygiene and decreased oral hygiene practices were observed during the pandemic compared with these practices in the prepandemic era. A similar tendency of changes in hand and oral hygiene before and during the pandemic was observed in the subgroup analysis. The trend in changes in hand and oral hygiene behaviors (prepandemic period [2008-2019] vs pandemic period [2020-2022]) is presented in eTables 2 and 3 in Supplement 1.

**Figure 3.**
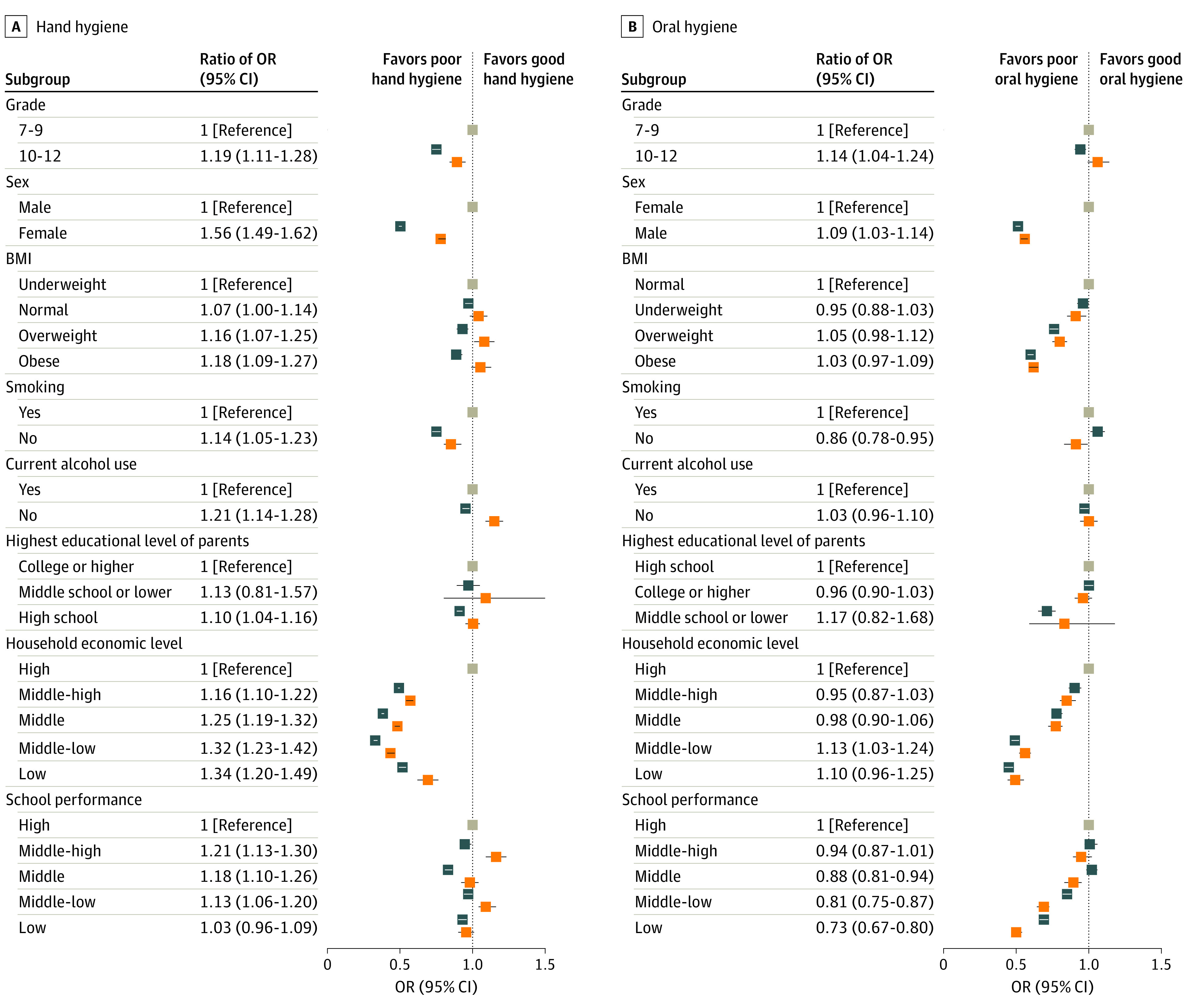
Ratio of Odds Ratios (ORs) for the Association Between Hand and Oral Hygiene Practices and Sociodemographic Factors For the non-reference values, blue indicates prepandemic data; orange indicates intrapandemic. BMI indicates body mass index (calculated as weight in kilograms divided by height in meters squared).

Figure 3 and eTables 4 and 5 in Supplement 1 detail the factors associated with hand and oral hygiene behavior. High school (ratio of OR, 1.19; 95% CI, 1.11-1.28), female sex (ratio of OR, 1.56; 95% CI, 1.49-1.62), overweight (ratio of OR, 1.16; 95% CI, 1.07-1.25), obesity (ratio of OR, 1.18; 95% CI, 1.09-1.27), nonsmoking status (ratio of OR, 1.14; 95% CI, 1.05-1.23), no alcohol consumption (ratio of OR, 1.21; 95% CI, 1.14-1.28), high school being the highest educational level of parents (ratio of OR, 1.10; 95% CI, 1.04-1.16), middle-high household economic level (ratio of OR, 1.16; 95% CI, 1.10-1.22), middle household economic level (ratio of OR, 1.25; 95% CI, 1.19-1.32), middle-low household economic level (ratio of OR, 1.32; 95% CI, 1.23-1.42), and low household economic level (ratio of OR, 1.34; 95% CI, 1.20-1.49) along with middle-high school performance (reference, high: ratio of OR, 1.21; 95% CI, 1.13-1.30), middle school performance (ratio of OR, 1.18; 95% CI, 1.10-1.26), and middle-low school performance (ratio of OR, 1.13; 95% CI, 1.06-1.20) were associated with improvement in hand hygiene behavior during the pandemic. Nonsmoking status (ratio of OR, 0.86; 95% CI, 0.78-0.95) along with middle (ratio of OR, 0.88; 95% CI, 0.81-0.94), middle-low (ratio of OR, 0.81; 95% CI, 0.75-0.87), and low (ratio of OR, 0.73; 95% CI, 0.67-0.80) school performance, female sex (ratio of OR, 0.92; 95% CI, 0.88-0.97), and midle-low household economic level (ratio of OR, 0.88; 95% CI, 0.81-0.97), were significantly associated with the risk of suboptimal oral hygiene during the pandemic compared with the prepandemic period.

## Discussion

### Study Findings

To the best of our knowledge, this is the first nationwide, long-term study to examine the 15-year trends in hand and oral hygiene using a data set of approximately 1 million South Korean adolescents. In addition, this study also analyzed the factors associated with hand and oral hygiene. We found that the estimated proportion of adolescents with an optimal level of handwashing decreased before the COVID-19 pandemic. However, as the pandemic began, the proportion increased and then subsequently decreased. Older age, female sex, nonsmoking status, current alcohol use, low household economic level, and poor school performance were significant risk factors for poor hand hygiene during the pandemic. Meanwhile, the estimated proportion of adolescents with good oral hygiene decreased steadily during the past 15 years, and this decrease became more prominent during the pandemic. Boys; nonsmokers; those with middle, middle-low, and low school performance; those without high household economic level; and those with underweight, overweight, or obesity had a higher risk of poor oral hygiene.

### Plausible Mechanism

This study indicated that the proportion of adolescents with good handwashing habits increased remarkably after the advent of COVID-19. The hands are critical vectors for transmitting infectious diseases.^23^ However, previous research supports the idea that handwashing was not considered a serious issue as it should have been.^24^ Throughout the pandemic, governments and health organizations emphasized handwashing education not only for those who worked in the health sector but also for the general public. Nationwide campaigns promoting handwashing appeared in the daily news, print ads, and billboards, which likely led to a noticeable growth in interest in hand hygiene. In addition, we found that various factors were associated with hand and oral hygiene practices. Despite exposure to the same campaigns, the degree of health awareness may vary, depending on individual circumstances and health literacy levels.^25^

We observed that the proportion of adolescents exceeding the recommended guidelines for toothbrushing decreased during the last 15 years, particularly during the pandemic. There are 2 possible explanations for this finding. First, during the pandemic, there were few campaigns related to toothbrushing, unlike for hand hygiene. Adolescents may have been more likely to neglect their oral health status than before, prioritizing the health of other organs that are directly linked to COVID-19. Studies on the association between toothbrushing and COVID-19 are scarce. Therefore, there is a lack of strong guidelines related to toothbrushing. Nonetheless, the oral cavity may contain pathogens that predispose individuals to viral infection, including COVID-19.^26^ Some studies have concluded that poor toothbrushing may increase the risk of COVID-19.^27,28^ Second, COVID-19–associated psychological distress may change oral health–related behaviors.^11,29^ Psychological distress caused by depression,^30^ loneliness, and suicidal ideation^31^ is known to be associated with a lower frequency of toothbrushing. Because social isolation increased during the pandemic lockdown, emotional distress increased, which may have negatively affected toothbrushing frequency.

### Comparison With Previous Studies

Few studies examined the association of the pandemic with hand or oral hygiene, and these studies were conducted in Nigeria,^10,11^ Denmark,^8^ and France.^9^ These studies showed conflicting results and a weak level of evidence because they were limited by small sample sizes, specific socioeconomic status, inappropriate statistical models, or short trends. Moreover, these studies conducted analyses over a wide age range. Importantly, we used a large population-based study with a data set that spanned 15 years to produce a representative sample and visualize long-term trends. Although another study conducted analyses over a wide range of ages, we focused specifically on adolescents because the formation of healthy hand and oral hygiene habits is important during adolescence and may subsequently be sustained into adulthood.^32^

### Policy Implications

Research has shown that although handwashing is a simple task for preventing the spread of infectious diseases, people tend to underestimate its importance.^33^ Because of the global endeavors to raise awareness about hand hygiene during the pandemic, there has been a significant increase in the frequency and quality of handwashing. We must ensure that this focus persists, even after the end of the pandemic. During the pandemic, campaigns using short videos on social media, poster advertisements, and mainstream television succeeded in promoting handwashing throughout the country. A previous study suggested that the promotion of hygiene behavior helps improve health outcomes and prevent the spread of infectious diseases.^34^ We should continue using platforms with high accessibility to reinforce awareness of hand hygiene practices. In addition, to maintain high hand hygiene adherence, the government should conduct training sessions on hand hygiene for adolescents, install handwashing stations with sanitizers in school, and organize awareness sessions for adolescents to help them understand the importance of preventing the spread of infections.^35^

Unlike in hand hygiene, this study highlighted a decrease in the frequency of toothbrushing among adolescents during the pandemic. Although research supports the association between toothbrushing and COVID-19, there was a lack of oral hygiene promotion as a preventive approach to COVID-19.^36^ The oral cavity is known to be a crucial site for SARS-CoV-2 replication^7^; however, the microbial pathway explaining its association has not been resolved yet.^37^ Moreover, because the transmission of COVID-19 occurs via droplets, it is crucial to keep the oral cavity clean with toothpaste that contains antiviral properties, including stannous fluoride, chlorine dioxide, and chlorhexidine.^38,39,40,41^ According to an official guideline provided by the Korean Academy of Preventive Dentistry, it is recommended and safe to use toothpaste with antimicrobial properties 3 to 4 times a day for both children and adults.^40^ Stannous fluoride is known to disrupt bacterial glycolysis and, when combined with fluoride, acts to deter the buildup of dental plaque and calculus on the tooth’s surface.^42^ Toothbrushing at least twice a day should be more strongly encouraged by dental professionals. We believe that oral hygiene should be regarded as an important approach for preventing SARS-CoV-2 infection.

### Strengths and Limitations

This study has several strengths. This is the first study, to our knowledge, to explore the long-term trends of hand and oral hygiene behaviors among Korean adolescents and identify how the pandemic is associated with this trend. We used 15 consecutive years from a representative sample of approximately 1 million respondents, which allowed for more comprehensive and elaborate analyses. Before the onset of the pandemic, there were few trend studies on hand and/or oral hygiene.^43^ This study enables us to explore trends before the COVID-19 pandemic because we conducted analyses spanning 3 years during the pandemic and 12 years of the prepandemic era. In addition to comparing the prepandemic and pandemic eras, this study also analyzed changes in hygiene behaviors throughout the overall pandemic era until the year 2022. Furthermore, we found that older age, current alcohol use, low household economic level, and poor school performance increased the risk of poor hand hygiene. This study reinforces the notion of improving handwashing campaigns that target those within the risk factor categories.

This study had a few limitations that need consideration. First, we used self-reported data to assess the frequency of handwashing and toothbrushing, which may have introduced recall and social desirability biases into our findings. Second, because the questionnaire responses regarding hand hygiene behaviors were on a 4-point Likert scale, we did not use quantitative measurements for hand hygiene behaviors. The responses may not have equal distances between them. Third, only Korean adolescents were included in the study, and the observed trends may differ from the global trends in toothbrushing and handwashing because the data reflect the socioeconomic context of South Korea. Fourth, because we are still amidst the pandemic and this study included data until 2022, there is a need to monitor the later era of the pandemic and identify changes in toothbrushing and handwashing habits.

## Conclusions

We investigated the 15-year trends in frequency of handwashing and toothbrushing and its associated factors using nationwide, large-scale data collected between 2008 and 2022. This study revealed an immediate increase in handwashing frequency at the beginning of the pandemic and a steady decrease thereafter. Meanwhile, oral hygiene practices did not show an immediate change but gradually decreased during the pandemic. Hence, this study recommends continued promotion of the importance of hygiene behaviors, even after the COVID-19 pandemic. Future studies should investigate this trend in the later era of the pandemic and identify its underlying mechanisms.

## Notes

Supplement 1. **eTable 1.** Interrupted Time Series Results for Change in Hand and Oral Hygiene Practices After Versus Before the COVID-19 Pandemic Among Korean Adolescents, 2008-2022 **eTable 2.** Adjusted and Weighted Estimation of Trend in Adolescent Hand Hygiene Practice in South Korea, 2008–2022 **eTable 3.** Adjusted and Weighted Estimation of Trend in Adolescent Oral Hygiene Practice in South Korea, 2008–2022 **eTable 4.** Adjusted and Weighted Logistic Regression Analysis for the Association Between Hand Hygiene Practice and Risk Factors **eTable 5.** Adjusted and Weighted Logistic Regression Analysis for the Association Between Oral Hygiene Practice and Risk Factors

Supplement 2.
Data Sharing Statement

## References

[1] Cucinotta D, Vanelli M. WHO declares COVID-19 a pandemic. Acta Biomed. 2020;91(1):157-160. doi:10.23750/abm.v91i1.9397 32191675 PMC7569573

[2] Kwon R, Rahmati M. Global, regional, and national COVID-19 vaccination rate in 237 countries and territories, March 2022: a systematic analysis for World Health Organization COVID-19 Dashboard, release 2. Life Cycle. 2022;2:e15. doi:10.54724/lc.2022.e15

[3] Park SH, Hong SH, Kim K, . Nonpharmaceutical interventions reduce the incidence and mortality of COVID-19: a study based on the survey from the International COVID-19 Research Network (ICRN). J Med Virol. 2023;95(2):e28354. doi:10.1002/jmv.28354 36447130 PMC9878143

[4] MacLeod C, Braun L, Caruso BA, . Recommendations for hand hygiene in community settings: a scoping review of current international guidelines. BMJ Open. 2023;13(6):e068887. doi:10.1136/bmjopen-2022-068887 37344109 PMC10314431

[5] Rosema N, Slot DE, van Palenstein Helderman WH, Wiggelinkhuizen L, Van der Weijden GA. The efficacy of powered toothbrushes following a brushing exercise: a systematic review. Int J Dent Hyg. 2016;14(1):29-41. doi:10.1111/idh.12115 25545231

[6] Treerutkuarkul A, Gruber K. Prevention is better than treatment. Bull World Health Organ. 2015;93(9):594-595. doi:10.2471/BLT.15.020915 26478621 PMC4581649

[7] Huang N, Pérez P, Kato T, ; NIH COVID-19 Autopsy Consortium; HCA Oral and Craniofacial Biological Network. SARS-CoV-2 infection of the oral cavity and saliva. Nat Med. 2021;27(5):892-903. doi:10.1038/s41591-021-01296-8 33767405 PMC8240394

[8] Stangerup M, Hansen MB, Hansen R, . Hand hygiene compliance of healthcare workers before and during the COVID-19 pandemic: a long-term follow-up study. Am J Infect Control. 2021;49(9):1118-1122. doi:10.1016/j.ajic.2021.06.014 34182068 PMC8233047

[9] Huang F, Armando M, Dufau S, Florea O, Brouqui P, Boudjema S. COVID-19 outbreak and healthcare worker behavioural change toward hand hygiene practices. J Hosp Infect. 2021;111:27-34. doi:10.1016/j.jhin.2021.03.004 33716086 PMC7948529

[10] Folayan MO, Ibigbami OI, Oloniniyi IO, Oginni O, Aloba O. Associations between psychological wellbeing, depression, general anxiety, perceived social support, tooth brushing frequency and oral ulcers among adults resident in Nigeria during the first wave of the COVID-19 pandemic. BMC Oral Health. 2021;21(1):520. doi:10.1186/s12903-021-01871-y 34645423 PMC8510883

[11] Folayan MO, Zuniga RAA, Ezechi OC, . Associations between emotional distress, sleep changes, decreased tooth brushing frequency, self-reported oral ulcers and SARS-Cov-2 infection during the first wave of the COVID-19 pandemic: a global survey. Int J Environ Res Public Health. 2022;19(18):11550. doi:10.3390/ijerph191811550 36141821 PMC9516999

[12] Kim Y, Choi S, Chun C, Park S, Khang YH, Oh K. Data resource profile: the Korea Youth Risk Behavior Web-based Survey (KYRBS). Int J Epidemiol. 2016;45(4):1076-1076e. doi:10.1093/ije/dyw070 27380796

[13] Kim MJ, Lee KH, Lee JS, . Trends in body mass index changes among Korean adolescents between 2005-2020, including the COVID-19 pandemic period: a national representative survey of one million adolescents. Eur Rev Med Pharmacol Sci. 2022;26(11):4082-4091. doi:10.26355/eurrev_202206_28978 35731079

[14] World Medical Association. World Medical Association Declaration of Helsinki: ethical principles for medical research involving human subjects. JAMA. 2013;310(20):2191-2194. doi:10.1001/jama.2013.281053 24141714

[15] Dighe A, Cattarino L, Cuomo-Dannenburg G, . Response to COVID-19 in South Korea and implications for lifting stringent interventions. BMC Med. 2020;18(1):321. doi:10.1186/s12916-020-01791-8 33032601 PMC7544529

[16] Addy M, Hunter ML, Kingdon A, Dummer PM, Shaw WC. An 8-year study of changes in oral hygiene and periodontal health during adolescence. Int J Paediatr Dent. 1994;4(2):75-80. doi:10.1111/j.1365-263X.1994.tb00108.x 7748854

[17] Kim JH, Yun S, Hwang SS, ; Committee for the Development of Growth Standards for Korean Children and Adolescents; Committee for School Health and Public Health Statistics, the Korean Pediatric Society; Division of Health and Nutrition Survey, Korea Centers for Disease Control and Prevention. The 2017 Korean National Growth Charts for children and adolescents: development, improvement, and prospects. Korean J Pediatr. 2018;61(5):135-149. doi:10.3345/kjp.2018.61.5.135 29853938 PMC5976563

[18] Woo HG, Park S, Yon H, . National trends in sadness, suicidality, and COVID-19 pandemic-related risk factors among South Korean adolescents from 2005 to 2021. JAMA Netw Open. 2023;6(5):e2314838. doi:10.1001/jamanetworkopen.2023.14838 37223902 PMC10209749

[19] Park S, Yon H, Ban CY, . National trends in alcohol and substance use among adolescents from 2005 to 2021: a Korean serial cross-sectional study of one million adolescents. World J Pediatr. 2023;19(11):1071-1081. doi:10.1007/s12519-023-00715-9 36977821 PMC10049906

[20] Raffa BJ, Schilling S, Henry MK, . Ingestion of illicit substances by young children before and during the COVID-19 pandemic. JAMA Netw Open. 2023;6(4):e239549. doi:10.1001/jamanetworkopen.2023.9549 37083660 PMC10122182

[21] Lee SW. Methods for testing statistical differences between groups in medical research: statistical standard and guideline of Life Cycle Committee. Life Cycle. 2022;2:e1. doi:10.54724/lc.2022.e1

[22] Lee SW. Regression analysis for continuous independent variables in medical research: statistical standard and guideline of Life Cycle Committee. Life Cycle. 2022;2:e3. doi:10.54724/lc.2022.e3

[23] Edmonds-Wilson SL, Nurinova NI, Zapka CA, Fierer N, Wilson M. Review of human hand microbiome research. J Dermatol Sci. 2015;80(1):3-12. doi:10.1016/j.jdermsci.2015.07.006 26278471

[24] Bezerra TB, Valim MD, Bortolini J, Ribeiro RP, Marcon SR, Moura MEB. Adherence to hand hygiene in critical sectors: can we go on like this? J Clin Nurs. 2020;29(13-14):2691-2698. doi:10.1111/jocn.15293 32301162

[25] Al-Dmour H, Masa’deh R, Salman A, Abuhashesh M, Al-Dmour R. Influence of social media platforms on public health protection against the COVID-19 pandemic via the mediating effects of public health awareness and behavioral changes: integrated model. J Med Internet Res. 2020;22(8):e19996. doi:10.2196/19996 32750004 PMC7439806

[26] Aas JA, Paster BJ, Stokes LN, Olsen I, Dewhirst FE. Defining the normal bacterial flora of the oral cavity. J Clin Microbiol. 2005;43(11):5721-5732. doi:10.1128/JCM.43.11.5721-5732.2005 16272510 PMC1287824

[27] Kamel AHM, Basuoni A, Salem ZA, AbuBakr N. The impact of oral health status on COVID-19 severity, recovery period and C-reactive protein values. Br Dent J. 2021:1-7. doi:10.1038/s41415-021-2656-1 33627848 PMC7904030

[28] Zhao SC, Li D. Immunologic classification of lymphocytic leukemia and its clinical significance. Article in Chinese. Zhonghua Nei Ke Za Zhi. 1988;27(7):429-431, 455. 3219949

[29] Matsuyama Y, Aida J, Takeuchi K, Koyama S, Tabuchi T. Dental pain and worsened socioeconomic conditions due to the COVID-19 pandemic. J Dent Res. 2021;100(6):591-598. doi:10.1177/00220345211005782 33792422 PMC8138328

[30] Anttila S, Knuuttila M, Ylöstalo P, Joukamaa M. Symptoms of depression and anxiety in relation to dental health behavior and self-perceived dental treatment need. Eur J Oral Sci. 2006;114(2):109-114. doi:10.1111/j.1600-0722.2006.00334.x 16630301

[31] Folayan MO, Tantawi ME, Oginni O, . Oral health practices and oral hygiene status as indicators of suicidal ideation among adolescents in Southwest Nigeria. PLoS One. 2021;16(2):e0247073. doi:10.1371/journal.pone.0247073 33630858 PMC7906320

[32] Gardner B, Lally P, Wardle J. Making health habitual: the psychology of ‘habit-formation’ and general practice. Br J Gen Pract. 2012;62(605):664-666. doi:10.3399/bjgp12X659466 23211256 PMC3505409

[33] Pittet D. Improving adherence to hand hygiene practice: a multidisciplinary approach. Emerg Infect Dis. 2001;7(2):234-240. doi:10.3201/eid0702.010217 11294714 PMC2631736

[34] Veys K, Dockx K, Van Remoortel H, Vandekerckhove P, De Buck E. The effect of hand hygiene promotion programs during epidemics and pandemics of respiratory droplet-transmissible infections on health outcomes: a rapid systematic review. BMC Public Health. 2021;21(1):1745. doi:10.1186/s12889-021-11815-4 34563144 PMC8467175

[35] Krishnamoorthy Y, Kala M, Kuberan D, Krishnan M, Tondare D. Compliance with hand hygiene practices and its appropriateness among healthcare workers during COVID-19 pandemic in public health facilities of Tamil Nadu, India. Heliyon. 2023;9(4):e15410. doi:10.1016/j.heliyon.2023.e15410 37089396 PMC10104598

[36] Addy M. Toothbrushing against coronavirus. Br Dent J. 2020;228(7):487. doi:10.1038/s41415-020-1450-9 32277179

[37] To KK, Tsang OT, Yip CC, . Consistent detection of 2019 novel coronavirus in saliva. Clin Infect Dis. 2020;71(15):841-843. doi:10.1093/cid/ciaa149 32047895 PMC7108139

[38] Akhter Y, Rastogi S, Kaithwas G. Frequent brushing of teeth inhibits the dissemination of the SARS-CoV-2: the biochemical mechanism. Environ Sustain (Singap). 2023;6:423-426. doi:10.1007/s42398-023-00279-4 37363087 PMC10226441

[39] Eduardo FP, Corrêa L, Mansur F, . Effectiveness of toothpastes on SARS-CoV-2 viral load in saliva. Int Dent J. 2022;72(6):825-831. doi:10.1016/j.identj.2022.03.006 35570015 PMC8940567

[40] Chung SY. Suggestions for preventive dental care guidelines according to age and oral health status. Int J Clin Prev Dent. 2020;16(2):45-50. doi:10.15236/ijcpd.2020.16.2.45

[41] Sheen S, Owens J, Addy M. The effect of toothpaste on the propensity of chlorhexidine and cetyl pyridinium chloride to produce staining in vitro: a possible predictor of inactivation. J Clin Periodontol. 2001;28(1):46-51. doi:10.1111/j.1600-051X.2001.280107.x 11142666

[42] Fiorillo L, Cervino G, Herford AS, Laino L, Cicciù M. Stannous fluoride effects on enamel: a systematic review. Biomimetics (Basel). 2020;5(3):41. doi:10.3390/biomimetics5030041 32878006 PMC7559150

[43] Honkala S, Vereecken C, Niclasen B, Honkala E. Trends in toothbrushing in 20 countries/regions from 1994 to 2010. Eur J Public Health. 2015;25(suppl 2):20-23. doi:10.1093/eurpub/ckv013 25805781

