# Supplementary material for: Hand and Oral Hygiene Practices of South Korean Adolescents Before and During the COVID-19 Pandemic
Source: JAMA Netw Open. 2023 Dec 26;6(12):e2349249. doi: 10.1001/jamanetworkopen.2023.49249 (PMC10751599; doi:10.1001/jamanetworkopen.2023.49249)
Supplement: Supplement 1. — eTable 1. Interrupted Time Series Results for Change in Hand and Oral Hygiene Practices After Versus Before the COVID-19 Pandemic Among Korean Adolescents, 2008-2022 eTable 2. Adjusted and Weighted Estimation of Trend in Adolescent Hand Hygiene Practice in South Korea, 2008–2022 eTable 3. Adjusted and Weighted Estimation of Trend in Adolescent Oral Hygiene Practice in South Korea, 2008–2022 eTable 4. Adjusted and Weighted Logistic Regression Analysis for the Association Between Hand Hygiene Practice and Risk Factors eTable 5. Adjusted and Weighted Logistic Regression Analysis for the Association Between Oral Hygiene Practice and Risk Factors [file jamanetwopen-e2349249-s001.pdf]

Supplementary Online Content

Oh J, Lee M, Lee H, et al. Hand and oral hygiene practices of South Korean adolescents before and during the COVID-19 pandemic. *JAMA Netw Open*. 2023;6(12):e2349249. doi:10.1001/jamanetworkopen.2023.49249

eTable 1. Interrupted Time Series Results for Change in Hand and Oral Hygiene Practices After Versus Before the COVID-19 Pandemic Among Korean Adolescents, 2008-2022

e**Table 2.** Adjusted and Weighted Estimation of Trend in Adolescent Hand Hygiene Practice in South Korea, 2008–2022

eTable 3. Adjusted and Weighted Estimation of Trend in Adolescent Oral Hygiene Practice in South Korea, 2008–2022

eTable 4. Adjusted and Weighted Logistic Regression Analysis for the Association Between Hand Hygiene Practice and Risk Factors

eTable 5. Adjusted and Weighted Logistic Regression Analysis for the Association Between Oral Hygiene Practice and Risk Factors

This supplementary material has been provided by the authors to give readers additional information about their work.

© 2023 Oh J et al. *JAMA Network Open.*

eTable 1. Interrupted Time Series Results for Change in Hand and Oral Hygiene Practices After Versus Before the COVID-19 Pandemic Among Korean Adolescents, 2008-2022

Subgroup		Parameter	OR (95% CI)	
			Hand hygiene	Oral hygiene
Overall		Intercept	**0.177 (0.171 to 0.183)**	**16.174 (15.540 to 16.834)**
		Time	**0.972 (0.969 to 0.976)**	**0.953 (0.950 to 0.957)**
		Immediate change	**1.952 (1.878 to 2.029)**	**1.119 (1.074 to 1.166)**
		Sustained change	**0.881 (0.860 to 0.902)**	**0.827 (0.807 to 0.847)**
Grade	7-9th	Intercept	**0.317 (0.287 to 0.350)**	**14.819 (12.741 to 17.234)**
		Time	**0.976 (0.972 to 0.981)**	**0.945 (0.940 to 0.950)**
		Immediate change	**1.855 (1.758 to 1.957)**	**1.069 (1.014 to 1.128)**
		Sustained change	**0.836 (0.809 to 0.864)**	**0.859 (0.833 to 0.886)**
	10-12th	Intercept	**0.266 (0.238 to 0.297)**	**16.951 (14.398 to 19.955)**
		Time	**0.970 (0.965 to 0.975)**	**0.961 (0.956 to 0.966)**
		Immediate change	**2.048 (1.938 to 2.165)**	**1.179 (1.106 to 1.256)**
		Sustained change	**0.941 (0.910 to 0.973)**	**0.796 (0.767 to 0.827)**
Sex	Male	Intercept	**0.243 (0.234 to 0.254)**	**12.123 (11.569 to 12.704)**
		Time	**0.970 (0.966 to 0.974)**	**0.953 (0.948 to 0.957)**
		Immediate change	**1.643 (1.569 to 1.722)**	**1.152 (1.095 to 1.211)**
		Sustained change	**0.907 (0.882 to 0.934)**	**0.844 (0.819 to 0.870)**
	Female	Intercept	**0.128 (0.120 to 0.135)**	**20.096 (18.718 to 21.576)**
		Time	**0.977 (0.972 to 0.983)**	**0.956 (0.949 to 0.962)**
		Immediate change	**2.539 (2.398 to 2.689)**	1.047 (0.975 to 1.124)
		Sustained change	**0.845 (0.818 to 0.873)**	**0.796 (0.764 to 0.829)**
BMI	Underweight	Intercept	**0.195 (0.179 to 0.214)**	**18.263 (16.200 to 20.590)**
		Time	**0.955 (0.946 to 0.963)**	**0.953 (0.941 to 0.965)**
		Immediate change	**2.038 (1.826 to 2.275)**	1.129 (0.981 to 1.299)
		Sustained change	**0.921 (0.860 to 0.986)**	**0.793 (0.730 to 0.861)**
	Normal	Intercept	**0.179 (0.173 to 0.186)**	**19.842 (18.978 to 20.745)**
		Time	**0.973 (0.969 to 0.976)**	**0.950 (0.946 to 0.955)**
		Immediate change	**1.968 (1.884 to 2.056)**	**1.149 (1.093 to 1.208)**
		Sustained change	**0.872 (0.850 to 0.896)**	**0.824 (0.800 to 0.850)**
	Overweight	Intercept	**0.171 (0.155 to 0.190)**	**15.156 (13.552 to 16.951)**
		Time	**0.983 (0.974 to 0.991)**	**0.953 (0.943 to 0.963)**
		Immediate change	**1.845 (1.679 to 2.027)**	**1.150 (1.032 to 1.282)**
		Sustained change	**0.896 (0.843 to 0.952)**	**0.822 (0.771 to 0.877)**
	Obese	Intercept	**0.157 (0.142 to 0.173)**	**12.064 (10.834 to 13.433)**
		Time	**0.980 (0.971 to 0.990)**	**0.973 (0.963 to 0.983)**
		Immediate change	**1.868 (1.712 to 2.039)**	0.956 (0.869 to 1.053)
		Sustained change	**0.883 (0.839 to 0.930)**	**0.849 (0.801 to 0.899)**

© 2023 Oh J et al. *JAMA Network Open.*

Smoking	Yes	Intercept	**0.278 (0.256 to 0.302)**	**12.518 (11.141 to 14.067)**
		Time	**0.937 (0.929 to 0.946)**	**0.984 (0.972 to 0.996)**
		Immediate change	**1.989 (1.749 to 2.262)**	1.164 (0.991 to 1.368)
		Sustained change	1.005 (0.923 to 1.094)	**0.765 (0.697 to 0.839)**
	No	Intercept	**0.152 (0.147 to 0.158)**	**17.148 (16.471 to 17.854)**
		Time	**0.976 (0.973 to 0.980)**	**0.950 (0.946 to 0.954)**
		Immediate change	**1.927 (1.852 to 2.005)**	**1.129 (1.082 to 1.178)**
		Sustained change	**0.873 (0.852 to 0.894)**	**0.832 (0.811 to 0.853)**
Current alcohol use	Yes	Intercept	**0.216 (0.202 to 0.230)**	**14.424 (13.367 to 15.565)**
		Time	**0.946 (0.940 to 0.952)**	**0.966 (0.959 to 0.974)**
		Immediate change	**2.095 (1.915 to 2.292)**	1.100 (0.990 to 1.223)
		Sustained change	**0.918 (0.867 to 0.972)**	**0.821 (0.769 to 0.876)**
	No	Intercept	**0.173 (0.166 to 0.180)**	**16.700 (15.909 to 17.532)**
		Time	**0.979 (0.976 to 0.983)**	**0.950 (0.946 to 0.954)**
		Immediate change	**1.890 (1.816 to 1.967)**	**1.132 (1.084 to 1.182)**
		Sustained change	**0.874 (0.853 to 0.896)**	**0.828 (0.808 to 0.850)**
Highest educational level of parents	Middle school or lower	Intercept	**0.206 (0.166 to 0.256)**	**12.594 (9.870 to 16.068)**
		Time	**0.951 (0.922 to 0.981)**	**0.950 (0.923 to 0.977)**
		Immediate change	**2.819 (1.637 to 4.854)**	1.239 (0.684 to 2.243)
		Sustained change	0.963 (0.646 to 1.437)	0.962 (0.628 to 1.475)
	High school	Intercept	**0.167 (0.158 to 0.176)**	**17.172 (16.058 to 18.366)**
		Time	**0.966 (0.959 to 0.972)**	**0.963 (0.956 to 0.971)**
		Immediate change	**2.465 (2.267 to 2.680)**	1.039 (0.935 to 1.154)
		Sustained change	**0.824 (0.777 to 0.873)**	**0.805 (0.750 to 0.864)**
	College or higher	Intercept	**0.185 (0.177 to 0.193)**	**22.045 (20.735 to 23.439)**
		Time	**0.973 (0.968 to 0.977)**	**0.934 (0.929 to 0.940)**
		Immediate change	**1.910 (1.811 to 2.015)**	**1.186 (1.114 to 1.263)**
		Sustained change	**0.869 (0.841 to 0.898)**	**0.843 (0.812 to 0.875)**
Household economic level	High	Intercept	**0.389 (0.356 to 0.426)**	**21.306 (17.738 to 25.590)**
		Time	**0.970 (0.963 to 0.976)**	**0.952 (0.938 to 0.966)**
		Immediate change	**1.555 (1.441 to 1.678)**	1.116 (0.977 to 1.276)
		Sustained change	**0.902 (0.860 to 0.946)**	**0.835 (0.773 to 0.901)**
	Middle-high	Intercept	**0.178 (0.166 to 0.190)**	**24.752 (22.151 to 27.658)**
		Time	**0.972 (0.967 to 0.977)**	**0.940 (0.933 to 0.947)**
		Immediate change	**1.928 (1.818 to 2.045)**	**1.184 (1.093 to 1.284)**
		Sustained change	**0.878 (0.846 to 0.910)**	**0.820 (0.781 to 0.860)**
	Middle	Intercept	**0.130 (0.124 to 0.136)**	**18.882 (17.821 to 20.003)**
		Time	**0.976 (0.971 to 0.980)**	**0.947 (0.942 to 0.952)**

© 2023 Oh J et al. *JAMA Network Open.*

		Immediate change	**2.048 (1.949 to 2.152)**	**1.146 (1.082 to 1.213)**
		Sustained change	**0.878 (0.852 to 0.905)**	**0.830 (0.803 to 0.858)**
	Middle-low	Intercept	**0.120 (0.111 to 0.129)**	**10.557 (9.833 to 11.335)**
		Time	**0.967 (0.959 to 0.975)**	**0.970 (0.962 to 0.977)**
		Immediate change	**2.351 (2.133 to 2.591)**	1.078 (0.980 to 1.185)
		Sustained change	**0.876 (0.821 to 0.934)**	**0.850 (0.798 to 0.905)**
	Low	Intercept	**0.180 (0.161 to 0.201)**	**8.694 (7.773 to 9.723)**
		Time	**0.959 (0.946 to 0.972)**	**0.980 (0.967 to 0.992)**
		Immediate change	**2.461 (2.061 to 2.937)**	0.902 (0.750 to 1.084)
		Sustained change	0.899 (0.798 to 1.013)	0.913 (0.807 to 1.033)
School performance	High	Intercept	**0.203 (0.186 to 0.222)**	**17.704 (15.543 to 20.166)**
		Time	**0.992 (0.985 to 0.998)**	**0.958 (0.948 to 0.968)**
		Immediate change	**1.512 (1.400 to 1.632)**	**1.222 (1.086 to 1.374)**
		Sustained change	**0.927 (0.883 to 0.973)**	**0.801 (0.747 to 0.860)**
	Middle-high	Intercept	**0.153 (0.142 to 0.165)**	**18.668 (17.105 to 20.373)**
		Time	**0.979 (0.974 to 0.985)**	**0.948 (0.941 to 0.955)**
		Immediate change	**1.924 (1.806 to 2.050)**	**1.228 (1.127 to 1.338)**
		Sustained change	**0.862 (0.828 to 0.898)**	**0.810 (0.770 to 0.853)**
	Middle	Intercept	**0.180 (0.170 to 0.192)**	**16.939 (15.688 to 18.291)**
		Time	**0.969 (0.964 to 0.975)**	**0.951 (0.944 to 0.958)**
		Immediate change	**2.095 (1.975 to 2.222)**	**1.126 (1.046 to 1.212)**
		Sustained change	**0.880 (0.847 to 0.914)**	**0.821 (0.786 to 0.858)**
	Middle-low	Intercept	**0.169 (0.159 to 0.179)**	**14.902 (13.954 to 15.914)**
		Time	**0.964 (0.959 to 0.969)**	**0.955 (0.948 to 0.961)**
		Immediate change	**2.133 (2.001 to 2.274)**	1.043 (0.968 to 1.123)
		Sustained change	**0.847 (0.813 to 0.882)**	**0.850 (0.814 to 0.888)**
	Low	Intercept	**0.184 (0.171 to 0.198)**	**11.952 (10.986 to 13.005)**
		Time	**0.959 (0.952 to 0.967)**	**0.955 (0.946 to 0.963)**
		Immediate change	**2.013 (1.833 to 2.212)**	1.019 (0.927 to 1.121)
		Sustained change	**0.909 (0.855 to 0.968)**	**0.838 (0.790 to 0.889)**

BMI, body mass index; OR, odds ratio

The bold numbers indicate a significant difference (P <0.05)

© 2023 Oh J et al. *JAMA Network Open.*

eTable 2. Adjusted and Weighted Estimation of Trend in Adolescent Hand Hygiene Practice in South Korea, 2008–2022

	Trend, β (95% CI)			
	Overall (2008-2023)	Before pandemic (2008-2019)	Intra pandemic (2019-2023)	Trend difference, β difference (95% CI)
Overall	0.023 (-0.004 to 0.050)	**-0.275 (-0.311 to -0.239)**	**0.330 (0.160 to 0.501)**	0.605 (-0.100 to 1.311)
Grade, n (%)				
7-9th (middle school)	0.008 (-0.033 to 0.048)	**-0.265 (-0.320 to -0.209)**	0.073 (-0.180 to 0.325)	**0.337 (0.031 to 0.644)**
10-12th (high school)	**0.037 (0.001 to 0.074)**	**-0.283 (-0.330 to -0.236)**	**0.580 (0.351 to 0.809)**	0.863 (-0.352 to 2.078)
Sex, n (%)				
Male	**-0.121 (-0.161 to -0.081)**	**-0.368 (-0.423 to -0.314)**	-0.086 (-0.328 to 0.155)	0.282 (-0.001 to 0.564)
Female	**0.203 (0.171 to 0.234)**	**-0.152 (-0.193 to -0.112)**	**0.814 (0.598 to 1.030)**	0.967 (-0.627 to 2.560)
BMI, mean (SD)				
Underweight	**-0.119 (-0.188 to -0.050)**	**-0.477 (-0.570 to -0.385)**	**0.666 (0.179 to 1.154)**	1.144 (-0.209 to 2.496)
Normal	0.012 (-0.018 to 0.042)	**-0.273 (-0.312 to -0.234)**	**0.294 (0.103 to 0.485)**	0.567 (-0.090 to 1.224)
Overweight	**0.135 (0.069 to 0.201)**	**-0.175 (-0.262 to -0.089)**	0.314 (-0.148 to 0.775)	0.489 (-0.210 to 1.188)
Obese	**0.146 (0.075 to 0.218)**	**-0.194 (-0.292 to -0.095)**	0.307 (-0.097 to 0.711)	0.501 (-0.318 to 1.320)
Smoking, n (%)				
Yes	**-0.454 (-0.545 to -0.363)**	**-0.807 (-0.924 to -0.691)**	**0.736 (0.062 to 1.410)**	1.543 (-0.044 to 3.130)
No	**0.061 (0.033 to 0.088)**	**-0.228 (-0.265 to -0.192)**	**0.306 (0.132 to 0.480)**	0.534 (-0.128 to 1.196)
Current alcohol use, n (%)				
Yes	**-0.299 (-0.356 to -0.242)**	**-0.565 (-0.635 to -0.494)**	0.278 (-0.090 to 0.646)	**0.843 (0.135 to 1.550)**
No	**0.090 (0.061 to 0.118)**	**-0.204 (-0.242 to -0.166)**	**0.337 (0.157 to 0.517)**	0.541 (-0.176 to 1.257)
Highest educational level of parents, n (%)				
Middle school or lower	**-0.228 (-0.448 to -0.008)**	**-0.452 (-0.706 to -0.198)**	0.945 (-2.006 to 3.897)	1.397 (-2.146 to 4.941)
High school	**-0.055 (-0.100 to -0.010)**	**-0.294 (-0.349 to -0.239)**	0.108 (-0.283 to 0.498)	0.402 (-0.067 to 0.870)
College or higher	-0.002 (-0.038 to 0.033)	**-0.288 (-0.336 to -0.240)**	0.176 (-0.057 to 0.408)	0.463 (-0.012 to 0.939)
Household economic level, n (%)				
High	**-0.233 (-0.326 to -0.139)**	**-0.567 (-0.695 to -0.439)**	0.270 (-0.250 to 0.790)	0.837 (-0.065 to 1.739)
Middle-high	0.025 (-0.019 to 0.069)	**-0.294 (-0.352 to -0.235)**	0.156 (-0.121 to 0.433)	**0.449 (0.010 to 0.889)**
Middle	**0.103 (0.072 to 0.133)**	**-0.184 (-0.224 to -0.144)**	**0.466 (0.264 to 0.669)**	0.651 (-0.302 to 1.603)
Middle-low	0.036 (-0.012 to 0.085)	**-0.231 (-0.292 to -0.170)**	**0.465 (0.071 to 0.858)**	0.696 (-0.208 to 1.599)
Low	-0.048 (-0.165 to 0.068)	**-0.417 (-0.564 to -0.271)**	0.405 (-0.660 to 1.471)	0.823 (-0.647 to 2.292)
School performance, n (%)				
High	**0.090 (0.024 to 0.156)**	**-0.152 (-0.238 to -0.066)**	**0.847 (0.419 to 1.276)**	0.999 (-0.652 to 2.651)

© 2023 Oh J et al. *JAMA Network Open.*

Middle-high	**0.086 (0.045 to 0.128)**	**-0.184 (-0.237 to -0.130)**	0.205 (-0.089 to 0.498)	0.388 (-0.258 to 1.034)
Middle	**0.057 (0.016 to 0.098)**	**-0.286 (-0.339 to -0.233)**	**0.449 (0.182 to 0.716)**	0.735 (-0.141 to 1.611)
Middle-low	**-0.042 (-0.082 to -0.002)**	**-0.326 (-0.379 to -0.273)**	-0.003 (-0.288 to 0.283)	**0.324 (0.035 to 0.612)**
Low	**-0.125 (-0.189 to -0.061)**	**-0.449 (-0.533 to -0.366)**	0.339 (-0.121 to 0.799)	0.788 (-0.073 to 1.649)

BMI, body mass index; CI, confidence interval.

The bold numbers indicate a significant difference ($P < 0.05$)

© 2023 Oh J et al. *JAMA Network Open.*

eTable 3. Adjusted and Weighted Estimation of Trend in Adolescent Oral Hygiene Practice in South Korea, 2008–2022

	Trend, β (95% CI)			
	Overall (2008-2023)	Before pandemic (2008-2019)	Intra pandemic (2019-2023)	Trend difference, β difference (95% CI)
Overall	-0.323 (-0.339 to -0.307)	-0.258 (-0.280 to -0.237)	-1.407 (-1.531 to -1.282)	-1.148 (-1.844 to -0.452)
Grade, n (%)				
7-9th (middle school)	**-0.381 (-0.403 to -0.360)**	**-0.303 (-0.332 to -0.274)**	**-1.381 (-1.551 to -1.211)**	**-1.078 (-1.321 to -0.834)**
10-12th (high school)	**-0.267 (-0.291 to -0.243)**	**-0.219 (-0.250 to -0.188)**	**-1.444 (-1.626 to -1.263)**	**-1.225 (-2.432 to -0.018)**
Sex, n (%)				
Male	**-0.385 (-0.410 to -0.360)**	**-0.332 (-0.364 to -0.299)**	**-1.615 (-1.805 to -1.425)**	**-1.283 (-1.522 to -1.044)**
Female	**-0.244 (-0.263 to -0.224)**	**-0.165 (-0.191 to -0.140)**	**-1.156 (-1.312 to -1.000)**	-0.990 (-2.577 to 0.596)
BMI, mean (SD)				
Underweight	**-0.329 (-0.380 to -0.278)**	**-0.251 (-0.320 to -0.182)**	**-1.360 (-1.775 to -0.945)**	-1.109 (-2.437 to 0.219)
Normal	**-0.301 (-0.318 to -0.283)**	**-0.253 (-0.276 to -0.230)**	**-1.239 (-1.380 to -1.098)**	**-0.986 (-1.630 to -0.342)**
Overweight	**-0.382 (-0.433 to -0.331)**	**-0.314 (-0.381 to -0.246)**	**-1.598 (-1.985 to -1.211)**	**-1.285 (-1.937 to -0.632)**
Obese	**-0.410 (-0.476 to -0.344)**	**-0.231 (-0.320 to -0.142)**	**-2.161 (-2.565 to -1.757)**	**-1.930 (-2.749 to -1.111)**
Smoking, n (%)				
Yes	**-0.173 (-0.241 to -0.105)**	**-0.107 (-0.196 to -0.018)**	**-1.631 (-2.130 to -1.132)**	**-1.524 (-3.045 to -0.004)**
No	**-0.334 (-0.350 to -0.318)**	**-0.271 (-0.292 to -0.249)**	**-1.394 (-1.520 to -1.267)**	**-1.123 (-1.774 to -0.472)**
Current alcohol use, n (%)				
Yes	**-0.258 (-0.296 to -0.220)**	**-0.200 (-0.248 to -0.153)**	**-1.416 (-1.734 to -1.099)**	**-1.216 (-1.898 to -0.534)**
No	**-0.336 (-0.353 to -0.320)**	**-0.272 (-0.294 to -0.249)**	**-1.404 (-1.535 to -1.274)**	**-1.133 (-1.838 to -0.427)**
Highest educational level of parents, n (%)				
Middle school or lower	**-0.415 (-0.616 to -0.213)**	**-0.458 (-0.698 to -0.218)**	**-0.277 (-2.903 to 2.348)**	0.180 (-3.096 to 3.457)
High school	**-0.265 (-0.297 to -0.232)**	**-0.205 (-0.245 to -0.165)**	**-1.532 (-1.881 to -1.183)**	**-1.327 (-1.761 to -0.893)**
College or higher	**-0.368 (-0.388 to -0.348)**	**-0.326 (-0.353 to -0.299)**	**-1.221 (-1.391 to -1.051)**	**-0.895 (-1.343 to -0.446)**
Household economic level, n (%)				
High	**-0.266 (-0.312 to -0.221)**	**-0.205 (-0.267 to -0.144)**	**-1.218 (-1.523 to -0.913)**	**-1.012 (-1.810 to -0.215)**
Middle-high	**-0.338 (-0.364 to -0.312)**	**-0.276 (-0.309 to -0.242)**	**-1.321 (-1.536 to -1.105)**	**-1.045 (-1.449 to -0.642)**
Middle	**-0.343 (-0.364 to -0.322)**	**-0.278 (-0.306 to -0.250)**	**-1.431 (-1.597 to -1.264)**	**-1.153 (-2.098 to -0.207)**
Middle-low	**-0.309 (-0.359 to -0.259)**	**-0.243 (-0.307 to -0.179)**	**-1.635 (-2.076 to -1.195)**	**-1.393 (-2.317 to -0.468)**
Low	**-0.294 (-0.400 to -0.189)**	**-0.183 (-0.316 to -0.051)**	**-1.912 (-2.933 to -0.891)**	**-1.729 (-3.166 to -0.291)**
School performance, n (%)				
High	**-0.237 (-0.274 to -0.200)**	**-0.198 (-0.248 to -0.149)**	**-1.069 (-1.358 to -0.779)**	-0.870 (-2.491 to 0.751)

© 2023 Oh J et al. *JAMA Network Open.*

Middle-high	**-0.284 (-0.311 to -0.257)**	**-0.248 (-0.283 to -0.212)**	**-1.160 (-1.374 to -0.945)**	**-0.912 (-1.526 to -0.298)**
Middle	**-0.307 (-0.333 to -0.280)**	**-0.242 (-0.277 to -0.208)**	**-1.325 (-1.527 to -1.122)**	**-1.082 (-1.941 to -0.224)**
Middle-low	**-0.373 (-0.406 to -0.340)**	**-0.285 (-0.328 to -0.241)**	**-1.601 (-1.859 to -1.343)**	**-1.316 (-1.577 to -1.055)**
Low	**-0.526 (-0.581 to -0.472)**	**-0.388 (-0.459 to -0.317)**	**-2.356 (-2.810 to -1.902)**	**-1.968 (-2.826 to -1.110)**

BMI, body mass index; CI, confidence interval.

The bold numbers indicate a significant difference ($P < 0.05$)

© 2023 Oh J et al. *JAMA Network Open.*

eTable 4. Adjusted and Weighted Logistic Regression Analysis for the Association Between Hand Hygiene Practice and Risk Factors

	Weighted OR (95% CI)			Ratio of OR
	Overall	Pre-pandemic	During pandemic	
Grade, n (%)				
7-9th (middle school)	1.0 (reference)	1.0 (reference)	1.0 (reference)	
10-12th (high school)	**0.74 (0.72 to 0.77)**	**0.75 (0.72 to 0.78)**	**0.89 (0.84 to 0.95)**	**1.19 (1.11 to 1.28)**
Sex, n (%)				
Male	1.0 (reference)	1.0 (reference)	1.0 (reference)	
Female	**0.55 (0.54 to 0.56)**	**0.50 (0.49 to 0.51)**	**0.78 (0.76 to 0.81)**	**1.56 (1.49 to 1.62)**
BMI, mean (SD)				
Underweight	1.0 (reference)	1.0 (reference)	1.0 (reference)	
Normal	0.98 (0.95 to 1.00)	**0.97 (0.95 to 1.00)**	1.04 (0.98 to 1.10)	1.07 (1.00 to 1.14)
Overweight	**0.96 (0.93 to 1.00)**	**0.93 (0.89 to 0.97)**	**1.08 (1.01 to 1.15)**	**1.16 (1.07 to 1.25)**
Obese	**0.95 (0.92 to 0.98)**	**0.89 (0.86 to 0.93)**	1.05 (0.99 to 1.13)	**1.18 (1.09 to 1.27)**
Smoking, n (%)				
Yes	1.0 (reference)	1.0 (reference)	1.0 (reference)	
No	**0.76 (0.74 to 0.79)**	**0.75 (0.72 to 0.78)**	**0.85 (0.80 to 0.92)**	**1.14 (1.05 to 1.23)**
Current alcohol use, n (%)				
Yes	1.0 (reference)	1.0 (reference)	1.0 (reference)	
No	1.00 (0.97 to 1.02)	**0.95 (0.93 to 0.98)**	**1.15 (1.09 to 1.21)**	**1.21 (1.14 to 1.28)**
Highest educational level of parents, n (%)				
College or higher	1.0 (reference)	1.0 (reference)	1.0 (reference)	
Middle school or lower	0.94 (0.86 to 1.02)	0.97 (0.89 to 1.05)	1.09 (0.80 to 1.50)	1.13 (0.81 to 1.57)
High school	**0.90 (0.88 to 0.92)**	**0.91 (0.89 to 0.93)**	1.00 (0.95 to 1.05)	**1.10 (1.04 to 1.16)**
Household economic level, n (%)				
High	1.0 (reference)	1.0 (reference)	1.0 (reference)	
Middle-high	**0.50 (0.49 to 0.51)**	**0.49 (0.48 to 0.50)**	**0.57 (0.54 to 0.59)**	**1.16 (1.10 to 1.22)**

© 2023 Oh J et al. *JAMA Network Open.*

Middle	**0.39 (0.39 to 0.40)**	**0.38 (0.37 to 0.39)**	**0.48 (0.46 to 0.50)**	**1.25 (1.19 to 1.32)**
Middle-low	**0.33 (0.32 to 0.34)**	**0.33 (0.32 to 0.34)**	**0.43 (0.41 to 0.46)**	**1.32 (1.23 to 1.42)**
Low	**0.51 (0.49 to 0.54)**	**0.52 (0.49 to 0.54)**	**0.69 (0.62 to 0.76)**	**1.34 (1.20 to 1.49)**
School performance, n (%)				
High	1.0 (reference)	1.0 (reference)	1.0 (reference)	
Middle-high	0.99 (0.96 to 1.01)	**0.95 (0.92 to 0.99)**	**1.16 (1.09 to 1.23)**	**1.21 (1.13 to 1.30)**
Middle	**0.86 (0.83 to 0.88)**	**0.83 (0.81 to 0.86)**	0.98 (0.92 to 1.04)	**1.18 (1.10 to 1.26)**
Middle-low	1.00 (0.97 to 1.02)	0.97 (0.94 to 1.00)	**1.09 (1.04 to 1.16)**	**1.13 (1.06 to 1.20)**
Low	**0.94 (0.91 to 0.96)**	**0.93 (0.90 to 0.96)**	0.96 (0.90 to 1.01)	1.03 (0.96 to 1.09)

BMI, body mass index; OR, odds ratio; SD, standard deviation.

The bold numbers indicate a significant difference ($P < 0.05$)

© 2023 Oh J et al. *JAMA Network Open.*

eTable 5. Adjusted and Weighted Logistic Regression Analysis for the Association Between Oral Hygiene Practice and Risk Factors

	Weighted OR (95% CI)			Ratio of OR
	Overall	Pre-pandemic	During pandemic	
Grade, n (%)				
7-9th (middle school)	1.0 (reference)	1.0 (reference)	1.0 (reference)	
10-12th (high school)	1.00 (0.96 to 1.04)	**0.94 (0.90 to 0.98)**	1.06 (0.99 to 1.14)	**1.14 (1.04 to 1.24)**
Sex, n (%)				
Female	1.0 (reference)	1.0 (reference)	1.0 (reference)	
Male	**0.53 (0.51 to 0.54)**	**0.51 (0.50 to 0.53)**	**0.56 (0.53 to 0.58)**	**1.09 (1.03 to 1.14)**
BMI, mean (SD)				
Normal	1.0 (reference)	1.0 (reference)	1.0 (reference)	
Underweight	**0.95 (0.91 to 0.98)**	**0.96 (0.92 to 1.00)**	**0.91 (0.85 to 0.98)**	0.95 (0.88 to 1.03)
Overweight	**0.75 (0.73 to 0.78)**	**0.76 (0.73 to 0.79)**	**0.80 (0.75 to 0.85)**	1.05 (0.98 to 1.12)
Obese	**0.58 (0.56 to 0.59)**	**0.60 (0.58 to 0.62)**	**0.62 (0.59 to 0.65)**	1.03 (0.97 to 1.09)
Smoking, n (%)				
Yes	1.0 (reference)	1.0 (reference)	1.0 (reference)	
No	1.02 (0.98 to 1.06)	**1.06 (1.01 to 1.11)**	**0.91 (0.83 to 0.99)**	**0.86 (0.78 to 0.95)**
Current alcohol use, n (%)				
Yes	1.0 (reference)	1.0 (reference)	1.0 (reference)	
No	**0.95 (0.92 to 0.98)**	0.97 (0.94 to 1.00)	1.00 (0.94 to 1.06)	1.03 (0.96 to 1.10)
Highest educational level of parents, n (%)				
High school	1.0 (reference)	1.0 (reference)	1.0 (reference)	
College or higher	**0.95 (0.93 to 0.98)**	1.00 (0.97 to 1.03)	0.96 (0.90 to 1.02)	0.96 (0.90 to 1.03)
Middle school or lower	**0.72 (0.67 to 0.78)**	**0.71 (0.65 to 0.77)**	0.83 (0.59 to 1.18)	1.17 (0.82 to 1.68)
Household economic level, n (%)				
High	1.0 (reference)	1.0 (reference)	1.0 (reference)	
Middle-high	**0.90 (0.87 to 0.94)**	**0.90 (0.86 to 0.95)**	**0.85 (0.80 to 0.91)**	0.95 (0.87 to 1.03)

© 2023 Oh J et al. *JAMA Network Open.*

Middle	**0.80 (0.77 to 0.83)**	**0.78 (0.75 to 0.82)**	**0.77 (0.72 to 0.82)**	0.98 (0.90 to 1.06)
Middle-low	**0.53 (0.50 to 0.55)**	**0.49 (0.47 to 0.52)**	**0.56 (0.52 to 0.60)**	**1.13 (1.03 to 1.24)**
Low	**0.48 (0.46 to 0.51)**	**0.45 (0.42 to 0.48)**	**0.49 (0.44 to 0.55)**	1.10 (0.96 to 1.25)
School performance, n (%)				
High	1.0 (reference)	1.0 (reference)	1.0 (reference)	
Middle-high	1.00 (0.97 to 1.04)	1.01 (0.97 to 1.06)	0.95 (0.89 to 1.02)	0.94 (0.87 to 1.01)
Middle	0.99 (0.95 to 1.02)	1.02 (0.98 to 1.06)	**0.89 (0.83 to 0.95)**	**0.88 (0.81 to 0.94)**
Middle-low	**0.82 (0.79 to 0.85)**	**0.85 (0.82 to 0.88)**	**0.69 (0.64 to 0.73)**	**0.81 (0.75 to 0.87)**
Low	**0.65 (0.63 to 0.68)**	**0.69 (0.66 to 0.72)**	**0.50 (0.47 to 0.54)**	**0.73 (0.67 to 0.80)**

BMI, body mass index; OR, odds ratio; SD, standard deviation.

The bold numbers indicate a significant difference (P <0.05)

© 2023 Oh J et al. *JAMA Network Open.*
