# Supplementary material for: Hand and Oral Hygiene Practices of South Korean Adolescents Before and During the COVID-19 Pandemic
Source: JAMA Netw Open. 2023 Dec 26;6(12):e2349249. doi: 10.1001/jamanetworkopen.2023.49249 (PMC10751599; doi:10.1001/jamanetworkopen.2023.49249)
Supplement: Supplement 2. — Data Sharing Statement [file jamanetwopen-e2349249-s002.pdf]

Data Sharing Statement

Oh. Hand and Oral Hygiene Practices of South Korean Adolescents Before and During the COVID-19 Pandemic. *JAMA Netw Open*. Published December 26, 2023. doi:10.1001/jamanetworkopen.2023.49249

Data
Data available: No

Additional Information
Explanation for why data not available: Data are available upon reasonable request. Study protocol, statistical code: available from DKY (yonkkang@gmail.com). Data set: available from the Korea Disease Control and Prevention Agency (KDCA) through data use agreement.
